# Genomic epidemiology of invasive Group A Streptococcus infections in Argentina, 2023: high prevalence of *emm*1-global and detection of *emm*1 hypervirulent lineages

**DOI:** 10.1128/spectrum.01310-24

**Published:** 2024-11-29

**Authors:** Lucía Cipolla, Ariel Gianecini, Tomas Poklepovich, Paula Etcheverry, Florencia Rocca, Mónica Prieto

**Affiliations:** 1 Servicio Bacteriología Especial, Instituto Nacional de Enfermedades Infecciosas, Ciudad Autónoma de Buenos Aires, Buenos Aires, Argentina; 2 Centro Nacional de Genómica y Bioinformática, Administración Nacional de Laboratorios e Institutos de Salud, Ciudad Autónoma de Buenos Aires, Buenos Aires, Argentina; Griffith University - Gold Coast Campus, Gold Coast, Queensland, Australia

**Keywords:** *Streptococcus pyogenes*, invasive microorganisms, hypervirulent lineages, molecular epidemiology, WGS, Argentina, South America, genomic epidemiology, *emm*1

## Abstract

The aim of this study is to describe the genomic epidemiology of invasive Group A Streptococcus (iGAS) in Argentina during 2023. A total of 476 invasive GAS isolates were subtyped and analyzed by whole-genome sequencing. A high prevalence of the *emm*1-type strain was observed. Among the *emm*1 strains, two highly virulent international variants, *emm*1-UK and *emm*1-DK, were identified. Additionally, a local variant, called *emm*1-ST1319, was detected. This seminal study provides initial insights into the genomic landscape of iGAS, contributing to active surveillance to monitor emerging virulent *emm*1 lineages and cross-border transmission in South America.

**IMPORTANCE:**

Amid the increase in invasive *Streptococcus pyogenes* infections in Argentina in 2023, the implementation of genomic analysis of isolates allowed us to detect at the national level the prevalence of the *emm*1 type and within the M1 type, emerging national and international hypervirulent lineages. These findings from our report shed light on the distribution of invasive Group A Streptococcus, laying the foundation for genomic surveillance of this pathogen.

## OBSERVATION

Group A Streptococcus (GAS) causes various human diseases, including pharyngitis, skin infections, invasive infections, and toxic shock syndrome (1). Invasive Group A Streptococcus (iGAS) is associated with high mortality globally (2).

The onset of the COVID-19 pandemic and the implementation of quarantine measures resulted in a global reduction in iGAS cases. This sharp drop in iGAS cases was followed by rapid expansion during 2022 and 2023 in Europe and North America (3). The magnitude and severity observed could be explained by the coincidence of the expansion of hypervirulent *emm*1 lineages and the decline in immunity of the human population to *Streptococcus pyogenes* (4). Furthermore, factors such as exposure history, underlying conditions, viral co-infection, and genetic susceptibility heighten susceptibility to *S. pyogenes* infection, but the role of strain-specific virulence is crucial (5).

In various European countries, the emergence of the *emm*1-UK lineage has been linked to invasive infections (6–9). Its rapid expansion seemingly displaces the contemporary epidemic *emm*1 iGAS, known as the *emm*1-global strain, which had a worldwide impact during the 1980 s (5). Notably, Denmark has reported the swift expansion of another *emm*1 lineage, *emm*1-DK, underscoring the significance of surveillance and identification of emerging and more virulent variants, particularly intra-*emm*1 lineages (10).

In December 2022, the Pan American Health Organization issued an alert regarding increasing iGAS cases in Uruguay (11), foreshadowing a similar rise in neighboring Argentina. By the end of 2022, Argentina witnessed significant increases in domestic iGAS cases, prompting the implementation of enhanced surveillance to actively track the introduction and circulation of international lineages through 2023 (12). Additionally, the detection of the *emm*1-ST1319 lineage encouraged the National Reference Laboratory (NRL) to track this emerging variant, initially in the southern provinces of our country and subsequently at the national level, to evaluate its potential to displace the *emm*1-global strain.

This study delves into the genomic epidemiology of iGAS in Argentina in 2023, shedding light on *emm*-type distribution and the emergence of hypervirulent *emm*1 lineages.

Between January and December 2023, the NRL received 476 isolates from across the country for genomic analysis. These isolates were obtained from cases of iGAS infections, which are reported as part of the mandatory national surveillance for this disease. IGAS cases were defined as any case of meningitis, pneumonia, sepsis, necrotizing fasciitis, streptococcal toxic shock syndrome, endocarditis, cellulitis, abscesses, septic arthritis, myositis, or other invasive infections where *S. pyogenes* is identified in a normally sterile site.

All isolates were subjected to whole-genome sequencing analysis. Briefly, each isolate was cultured on blood agar base plates, and the species identification was confirmed by MALDI-TOF MS (Bruker Daltonics). Genomic DNA was extracted using the QIAMP DNA mini kit (QIAGEN) according to the manufacturer’s recommendations. DNA concentration was quantified using the Invitrogen Qubit assay (Thermo Fisher Scientific Inc., Waltham, MA, USA), and DNA samples were stored at −20°C until further processing. A multiplexed sequencing library was prepared with Nextera XT DNA (Illumina) and sequenced on the MiSeq platform (Illumina) to generate 250-bp paired-end reads, aiming for an expected coverage depth of >50× for each isolate.

Sequence quality assessment was performed using FastQC 0.11.9 (http://www.bioinformatics.babraham.ac.uk/projects/fastqc/), and contaminants were identified using Kraken2 v2.0.6 (Johns Hopkins University, http://ccb.jhu.edu/software/kraken2/). *De novo* assemblies were generated using Unicycler v.0.4.8 (13). Assembly quality assessment was performed using Quast v5.0.2. The average number of contigs was 34, and the average N50 contig length was 169535. *Emm* type and multi-locus sequence type (ST) were identified using CDC (https://www2.cdc.gov/vaccines/biotech/strepblast.asp) and PubMLST (https://pubmlst.org/organisms/streptococcus-pyogenes) databases. Exotoxin genes (*spe*A to *spe*C, *spe*G to *spe*M, *ssa*, *sme*Z) were obtained through the VFDB database (www.mgc.ac.cn/VFs/) with ARIBA (14).

*Emm*1 genomes were used in phylogenetic analysis. Single nucleotide polymorphism (SNP) calling was performed with Snippy v4.4.5 (https://github.com/tseemann/snippy) using complete *emm*1.0 MGAS5005 genome (GenBank accession number CP000017) as the reference. Moreover, representative isolates of *emm*1-global (*n* = 4), *emm*1-UK (*n* = 5), and *emm*1-DK (*n* = 1) clones were selected from public repositories and included in the analysis.

Recombinant regions were identified and filtered using Gubbins v3.2.1 (15), and the resulting core SNP alignment consisted of 785 sites. A maximum likelihood tree was inferred using IQTREE v1.6.1 with 10,000 bootstrap replicates under the nucleotide substitution model GTR+I+G. Phylogenetic trees were visualized using FigTree v1.4.2 (https://evomics.org/resources/software/molecular-evolution-software/figtree/).

*Emm1* strain data (geographical, pediatric, or adult origin, isolation source, month of isolation, and fatal outcome) and exotoxin profiles are available (https://microreact.org/project/n3ya9E7JYZQKH6oRdMEMHe-igasm1argentina2023).

A total of 476 isolates were analyzed, revealing a prevalence of the *emm*1 type at 57% (*n* = 274), followed by *emm*12 at 17% (*n* = 80), *emm*87 at 3.5% (*n* = 17), *emm*3 at 3.5% (*n* = 17), *emm*6 at 3% (*n* = 14), *emm*49 at 3% (*n* = 13), and *emm*4 at 3% (*n* = 13). *Emm* types with less than 10 isolates collectively accounted for 10% (*n* = 48).

Among the 274 *emm*1 iGAS cases, the sources of isolates were as follows: blood (58%, *n* = 158), skin and soft tissues (19%, *n* = 52), lower respiratory tract (8%, *n* = 21), and other body sites (15%, *n* = 43).

Within the *emm*1 isolates, the distribution of lineages was as follows: *emm*1-global 220/274 (78%), *emm*1-UK 30/274 (12%), *emm*1-ST1319 22/274 (9%), and *emm*1-DK 2/274 (1%). The lineage *emm*1-ST1319 was associated with the highest mortality rate (7/22, 33%), followed by *emm*1-UK (7/30, 24%) and *emm*1-global (30/220, 14%). No significant associations between *emm*1 lineages and pediatric or adult groups were observed. Severe infections occurred at a slightly higher rate in pediatric patients (55%) than in adults (45%).

**Fig 1:**
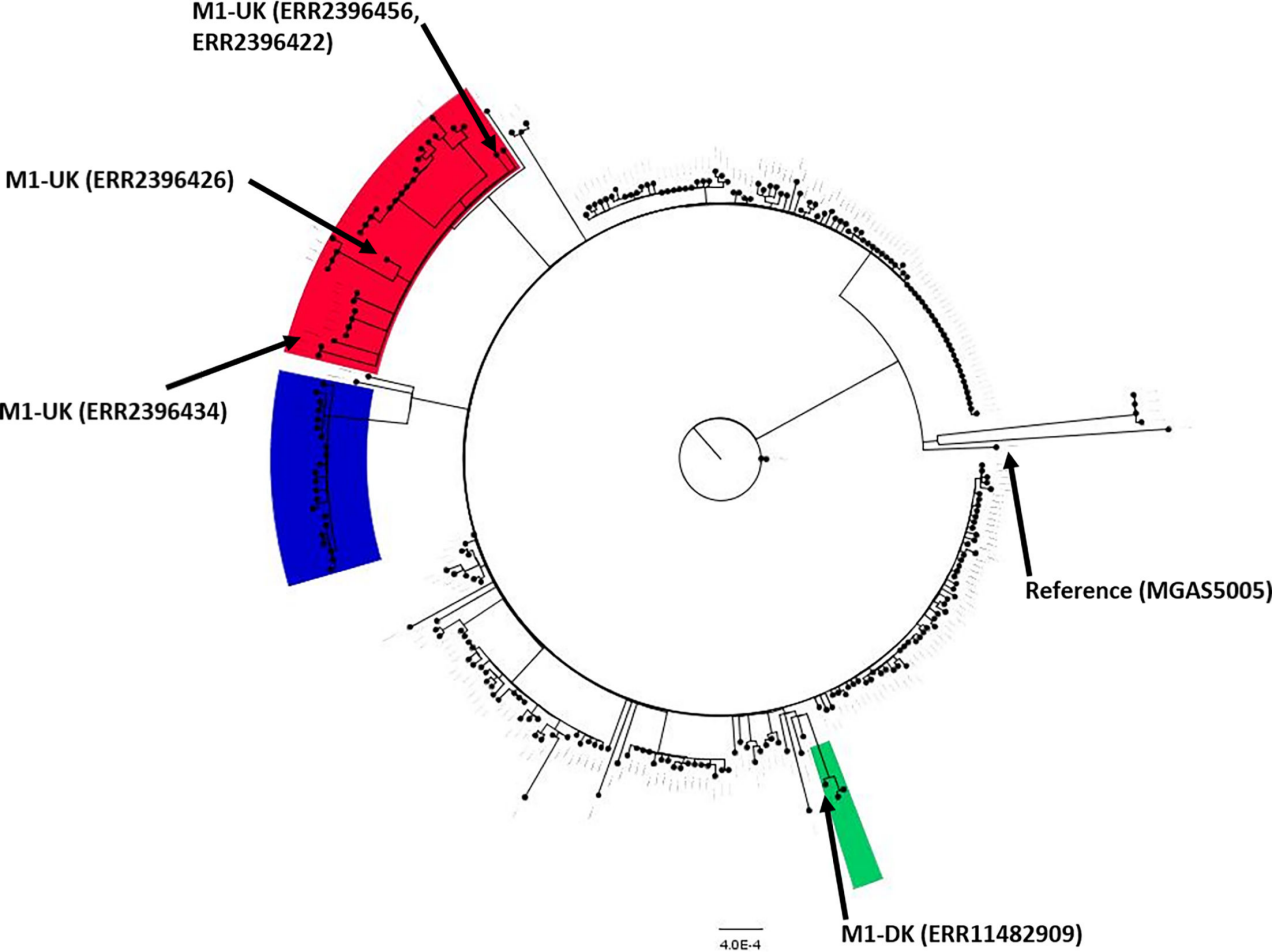
Maximum likelihood phylogenetic tree constructed from core single-nucleotide polymorphisms of 274 *emm*1 isolates. Clades corresponding to the M1-UK, M1-DK, and M1-ST1319 lineages are colored in red, green, and blue, respectively. Reference genomes for selected *emm*1-global, *emm*1-UK, *emm*1-DK are annotated included in the tree. The scale bar indicates the nucleotide substitutions per site.

**Fig 2:**
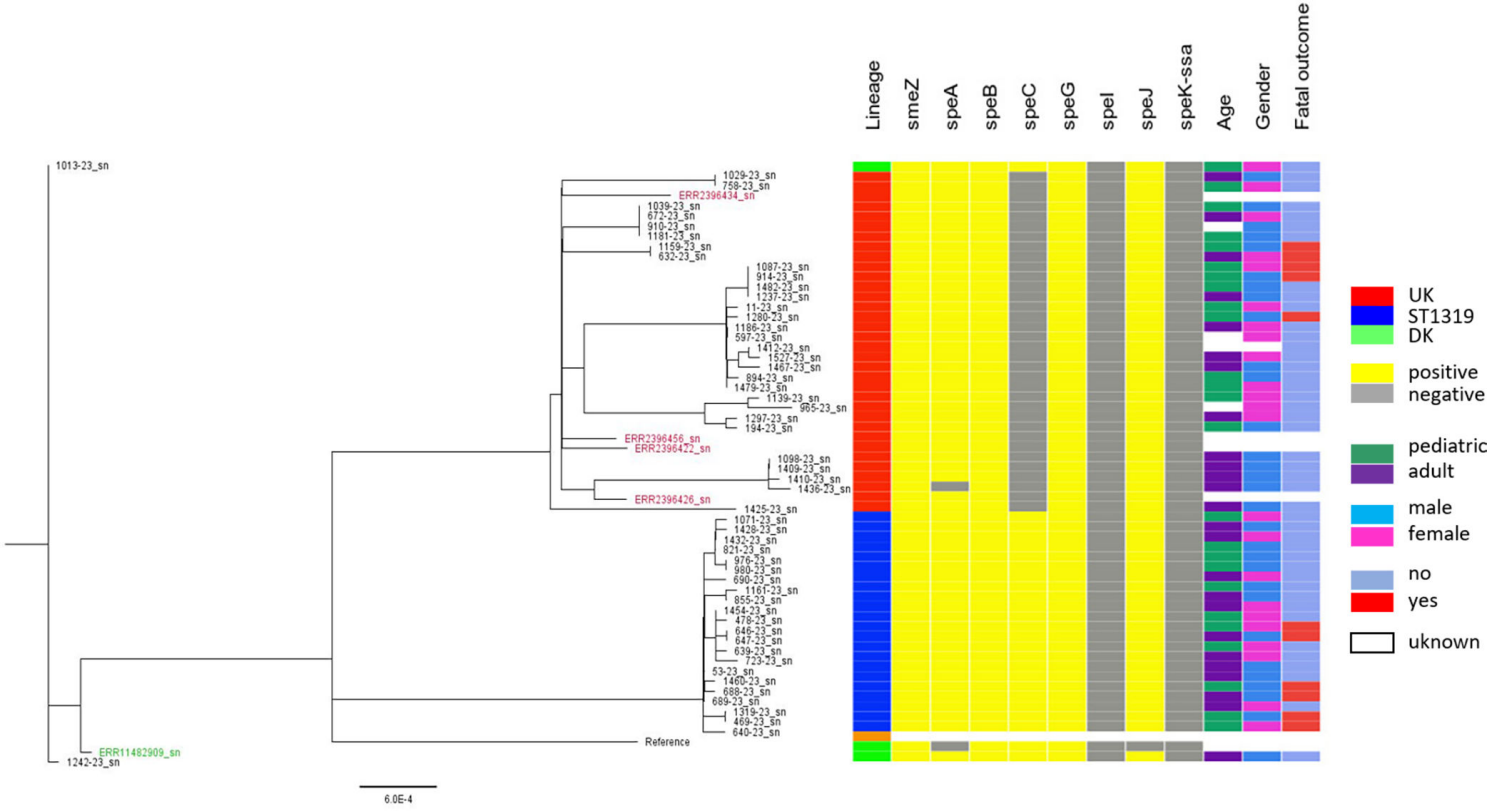
Maximum likelihood phylogenetic tree constructed from core single-nucleotide polymorphisms of M1-UK (*n* = 30), M1-DK (*n* = 2), and M1-ST1319 (*n* = 22) lineage isolates to the strain. Labels indicate isolate identity. Red and green colors indicate internacional representative isolates belonging to M1-UK and M1-DK lineages, respectively. The following columns describe exotoxin profiles, age, gender, and fatal outcome.

*Emm*1 phylogenetic tree showed distinct clades from intra*-emm*1 lineages, like *emm*1-UK and *emm*1-DK (Fig. 1). *Emm*1-UK isolates possessed all 27 SNPs across the core genome that characterized this lineage. No intermediate *emm*1-UK isolates (between 13 and 23 of the unique SNPs) were detected. More genomic diversity was observed among *emm*1-global strains. Isolates, identified as *emm*1-ST1319 by MLST analysis, formed a distinct phylogenetic clade (Fig. 1) in SNP analyses compared with other *emm*1 lineages. *Emm*1-UK strains had the same exotoxin profile: smeZ +, speA +, speB +, speC −, speG +, speH −, speI −, speJ +, speK −, speL −, speM −, ssa −. *Emm*1-ST1319 strains were characterized by the acquisition of the speC exotoxin, and all strains were characterized by the same exotoxin profile: smeZ +, speA +, speB +, speG +, speH −, speI −, speJ +, speK −, speL −, speM −, ssa − (Fig. 2). And *emm*1-DK had the following exotoxin repertoire: smeZ +, speA +, speB +, speC +, speG +, speH −, speI −, speJ +, speK −, speL −, speM −, ssa − (Fig. 2).

Sequence reads of Argentinian *emm*1-UK, *emm*1-DK, and *emm*1-ST1319 isolates are available from the European Nucleotide Archive (ENA) database under accession number PRJEB73573.

Several European and North American countries have reported iGAS upsurges in the period 2022–2023, which have been chronologically associated with the end of mitigation strategies during COVID-19 pandemic, including association with *emm*1 lineages. In Argentina, the sudden and unexpected increase in iGAS cases during 2023, along with the PAHO alert, prompted the NRL to transition from *emm* genotyping by the Sanger method to whole-genome sequencing for iGAS surveillance. This transition not only provided insights into the distribution of *emm* types but also enabled the detection of the genetic content and the introduction of hypervirulent iGAS lineages described worldwide.

Based on a comprehensive analysis of genomic data from *S. pyogenes* isolates obtained during the study period in Argentina, several significant conclusions can be drawn. Firstly, there was a high prevalence of the *emm*1 strain compared to other *emm* types, underscoring its significance in invasive infections in our country. The *emm*12 type emerged as the second most represented *emm* type among the iGAS isolates.

Within *emm*1 *S. pyogenes* strains, the highest proportion was identified as the *emm*1-global lineage. Phylogenetic and SNP analysis revealed the presence of different lineages. Two emerging international highly virulent *emm*1 variants, *emm*1-UK and *emm*1-DK, were identified. Nonetheless, these were represented in a low proportion, less than 12% each. The NRL continues to monitor the behavior of both international lineages and analyze whether or not they displace the *emm*1-global clone. The introduction and detection of these lineages in our epidemiological scenario highlight the importance of monitoring the spread of international clones and the need for active and ongoing surveillance to evidence the importation of these strains both at a national and regional level.

In addition, a local *S. pyogenes emm*1 lineage, designated *emm*1-ST1319 based on its MLST profile, was identified. This clone possesses the speC exotoxin gene, similar to *emm*1-DK clone, but lacks the characteristic SNPs associated with this lineage. Until September 2023, 72% of *emm*1-ST1319 cases were reported in the Patagonian provinces, consistent with regional transmission. Then, this clone was subsequently spread to other provinces. The average number of mutations between *emm*1-ST1319 isolates from the same clade ranged from 0 to 6 SNPs. Further genomic studies are warranted to fully characterize and determine the behavior and prevalence of *emm*1-ST1319 clones, as the acquisition of the *spe*C exotoxin gene may confer a selective advantage, potentially leading to more severe infections or distinct clinical characteristics compared to other *emm*1 lineages. Both *emm*1-ST1319 and *emm*1-UK have been associated with higher mortality rates compared with *emm*1-global.

The capacity of *S. pyogenes* to produce epidemic waves is multifactorial; however, genomic epidemiological approaches are essential to understand the mechanism underlying the evolutionary genomics of these epidemics. In Argentina, the iGAS case upsurge in 2023 was triggered by the *emm*1-global expansion over *emm*1-UK, *emm*1-DK, and *emm*1-ST1319 lineages.

Recently, Vieira and col (15). conducted a phylodynamic analysis on *emm*1 lineages, using a large genome data set that included *emm*1-global and *emm*1-UK from high-income countries, but no inferences could be addressed about importation and behavior of *emm*1-UK in some low-income countries because of the lack of information. Genomic surveillance of iGAS is not performed in any South American country. This study is the first to integrate genomic analysis into the surveillance of this pathogen, providing molecular characterization of iGAS infections during their emergence in 2023 and facilitating the identification and tracking of emerging lineages. Overall, this work provides crucial insights into the genomic epidemiology of invasive *S. pyogenes* in Argentina and lays the groundwork for future research and infectious disease control strategies.
